# Nutrients from spawning salmon influence leaf area, tissue density, and nitrogen‐15 in riparian plant leaves

**DOI:** 10.1002/ece3.11041

**Published:** 2024-02-20

**Authors:** Allison M. Dennert, Elizabeth Elle, John D. Reynolds

**Affiliations:** ^1^ Department of Biological Sciences Simon Fraser University Burnaby British Columbia Canada

**Keywords:** leaf area, marine‐derived nutrients, plant traits, salmon, subsidy

## Abstract

Nutrient subsidies have significant impacts on ecosystems by connecting disjunct habitats, often through long‐distance animal migrations. Salmon migrations on the North Pacific coasts provide these kinds of nutrient subsidies from senescent fish at the end of their life cycle, which can have significant ecological effects on terrestrial species. This can include impacts on individuals, populations, and communities, where shifts in community composition towards plant species that indicate nitrogen‐rich soils have been documented. We investigated the effects of variation in salmon spawning density on the leaf traits of four common riparian plant species on the central coast of British Columbia, Canada. We found that all plant species had higher foliar salmon‐derived nitrogen on streams with a higher spawning density. Three of the four species had larger leaves, and one species also had higher leaf mass per area on streams with more salmon. However, we found no differences in leaf greenness or foliar percent nitrogen among our study streams. These results demonstrate that nutrient subsidies from spawning salmon can have significant impacts on the ecology, morphology, and physiology of riparian plants, which lends support to a mechanism by which certain plants are more common on productive salmon streams.

## Graphical abstract

Salmon migrations on the North Pacific coasts provide these kinds of nutrient subsidies from senescent fish at the end of their life cycle, which can have significant ecological effects on terrestrial species, such as plants. We found that multiple plant species had larger leaves and higher foliar salmon‐derived nitrogen on streams with a higher spawning salmon density.

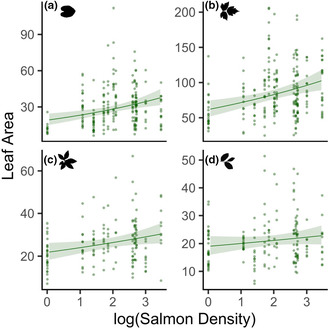

## 1 INTRODUCTION

Nutrient subsidies can have profound impacts on recipient ecosystems (Polis & Hurd, 1996). Subsidies can connect distant and disparate ecosystems through long‐distance animal migrations (Loreau et al., 2003), or adjacent ecosystems through deposition of nutrients nearby. These subsidies can happen annually and predictably, such as autumn leaf litter collected in streams (Richardson et al., 2010). They can also be unpredictable and stochastic, such as whale falls that deposit marine detritus on the ocean floor (Smith et al., 2015). Subsidies can have ecological impacts on a variety of scales, from individual organisms and populations to communities and whole ecosystems (Gounand et al., 2018). For example, subsidies can alter individual physiology and behavior (van den Top et al., 2018; Wilcox et al., 2021), population abundance (Field & Reynolds, 2011; Wagner & Reynolds, 2019), community composition (Hocking & Reynolds, 2011; Hurteau et al., 2016), and ecosystem function (Subalusky et al., 2015). Subsidies can even amend fundamental ecological theory, such as the intersection between subsidies and Island Biogeography Theory (Anderson & Wait, 2001; Obrist et al., 2022).

The coasts of the North Pacific receive pulsed nutrient subsidies during annual Pacific salmon (*Oncorhynchus* spp.) spawning migrations (Naiman et al., 2002; Schindler et al., 2003). After rearing in freshwater, salmon grow for several years at sea before returning to their natal streams to spawn and die. Chum (*O*. keta) and pink salmon (*O. gorbuscha*), in particular, spend the shortest amount of time in freshwater of any salmon species (Quinn, 2018). As such, these species gain nearly all of their body mass in the ocean and deliver these nutrients to coastal ecosystems upon their return, where flooding and large predators bring these nutrients onto land (Ben‐David et al., 1998; Darimont et al., 2010; Harding et al., 2019). Subsidies from spawning Pacific salmon have been shown to influence a variety of taxa, from plants (Ben‐David et al., 1998; Hocking & Reynolds, 2011), to aquatic and terrestrial invertebrates (Hocking et al., 2009, 2013), to birds (Christie & Reimchen, 2008; Field & Reynolds, 2011). The effects of salmon subsidies are varied (reviewed by Walsh et al., 2020), ranging from organismal abundance and behavior (Wilcox et al., 2021) to community composition (Hurteau et al., 2016), phenology (Lisi & Schindler, 2011), and even reproductive effort (Dennert et al., 2023). Plant communities are particularly affected by nutrient subsidies, as they intersect the abiotic and biotic components of ecosystems. Prior work on riparian plants in salmon ecosystems has shown evidence for increased growth (Quinn et al., 2018), reproductive output (Siemens et al., 2020), capacity for gas exchange (van den Top et al., 2018), and phylogenetic dispersion leading to compositional shifts towards high soil nitrogen indicator species (Hocking & Reynolds, 2011; Hurteau et al., 2016). While these patterns are well‐documented, little work has been done to date that contributes to a mechanistic understanding of these patterns in riparian plant communities. In particular, the physiological and morphological foundations of these compositional shifts in riparian communities remain unexplored.

Mineral and nutrient availability can affect plant morphology, physiology, and community composition in a variety of ways (Chapin, 1980). Nitrogen and phosphorous deposition can result in morphological changes through increased investment in plant growth, vegetative structures, root spread, and reproduction (Marschner, 1995). Plant physiology may be altered by nutrient availability through increases in RuBisCO and chlorophyll *a* production (Ågren, 2008), as well as changes in leaf tissue and stomatal density (Poorter et al., 2009). Lastly, plant communities along nitrogen gradients show varied patterns of succession and dominance, and nitrogen supply can directly influence the strength of interspecific competition (Tilman, 1987). Here, we examine some of the potential mechanisms that may be underpinning shifts in plant community composition in riparian areas on salmon streams.

We tested for differences in plant traits across 14 watersheds that vary in spawning salmon density on the central coast of British Columbia, Canada. We aimed to test whether nutrient inputs due to variation in salmon spawning density affect leaf nitrogen, leaf mass per area, leaf greenness, and leaf area in four common species of riparian plant that vary in their preference for nitrogen and soil fertility. We selected each of these leaf characteristics due to their role in photosynthesis, as exploring these traits may help mechanistically explain why some nitrogen indicator plants are found more commonly on streams with more salmon (Hocking & Reynolds, 2011; Hurteau et al., 2016). Photosynthetic capacity can be thought of as a relationship between CO_2_ supply and demand (Hultine & Marshall, 2000; Poorter et al., 2009). Stomata regulate supply of CO_2_ to the chloroplasts, nitrogen represents demand in the chloroplasts by chlorophyll *a* and RuBisCO, and leaf mass per area represents the intermediate mesophyll tissue between CO_2_ supply and demand (Hultine & Marshall, 2000). Thus, measuring leaf nitrogen and leaf mass per area will help us understand how these species are responding to changes in available soil nitrogen. Measuring greenness and leaf area and their relationship to spawning salmon density will tell us whether these plant species are producing more chloroplasts per unit area, or simply more leaf area overall in response to nutrient inputs.

We predicted that plants growing in watersheds with higher salmon density would have higher leaf nitrogen content, both in the marine‐derived isotope nitrogen‐15 and in their percent nitrogen content (Hocking & Reynolds, 2011, 2012; Mathewson & Reimchen, 2003; Wilkinson et al., 2005). Next, building on prior work indicating that one of our study species has more leaf stomata in areas of high salmon spawning density (van den Top et al., 2018), we predicted that plants on the banks of salmon‐dense watersheds would have an elevated photosynthetic capacity or rate, which would be seen in higher leaf greenness (Brown et al., 2020; Hultine & Marshall, 2000; Kieran et al., 2021) and larger leaf area (Dennert et al., 2023). Larger leaf area may be coupled with a lower leaf mass per area as is commonly seen in fertile soils (Poorter et al., 2009). Lastly, we hypothesized that nitrogen indicator species would have a stronger response to salmon density and marine‐derived nitrogen, as this may explain their higher proportional abundance on streams with more spawning salmon (Hurteau et al., 2016).

## 2 METHODS

### 2.1 Study area

We studied the relationship between spawning salmon and riparian plants in 14 watersheds in Haíɫzaqv (Heiltsuk) Territory on the central coast of British Columbia, Canada. These 14 watersheds are accessible only by boat from the town of Wágḷísḷa (Bella Bella; 52°9′46.858″ N, 128°8′35.442″ W), which is in the Coastal Western Hemlock Biogeoclimatic zone. This region receives over 3000 mm of rainfall annually (Pojar et al., 1987), and has historically had strong runs of Pacific salmon (*Oncorhynchus* spp.). Our study area is dominated by chum (*O. keta*) and pink salmon (*O. gorbuscha*), which spawn in September and October. All except one of our study streams have water flow driven by precipitation (rather than snow melt), and adjacent riparian soils in this biogeoclimatic region are generally nitrogen‐ and phosphorous‐poor (Prescott et al., 1993; Reimchen et al., 2002).

We selected four common species of perennial riparian plant: two nitrogen indicator species and two ericaceous species. Nitrogen indicator species were false lily‐of‐the‐valley (*Maianthemum dilatatum*; Family Asparagaceae) and salmonberry (*Rubus spectabilis*; Family Rosaceae). These species thrive in nutrient‐rich soils, and are known to grow more frequently on streams with a high abundance of spawning salmon (Douglas et al., 1999a, 2009; Hocking & Reynolds, 2011; Klinka et al., 1989). Ericaceous species were false azalea (*Menziesia ferruginea*; Family Ericaceae) and two blueberry species that were pooled due to difficulty in distinguishing between them in the absence of flowers or fruits (Pojar & MacKinnon, 1994): *Vaccinium ovalifolium* and *V. alaskaense*; Family Ericaceae. Ericaceous species are generally found in soils with poor or average nutrient availability, and false azalea has been shown to be more common on streams with a lower abundance of spawning salmon (Douglas et al., 1999b; Hocking & Reynolds, 2011; Klinka et al., 1989).

### 2.2 Salmon surveys

Our study watersheds ranged in salmon abundance from no salmon returning to tens of thousands of fish returning. We calculated spawning salmon densities using 3 years of data from long‐term monitoring in the region, as well as data from the Haíɫzaqv (Heiltsuk) First Nation and Fisheries and Oceans Canada. Each stream was surveyed for chum and pink salmon up to three times during the spawning season. Live and dead salmon were counted from the estuary up until a spawning barrier, or until no fish were observed. Estimates for annual salmon abundance were derived either using the area under the curve estimate (English et al., 1992) (for streams that were enumerated three times in a season) or the peak live + dead estimate (for streams that were enumerated one or two times in a season). The two methods yield very similar population estimates (Hocking & Reynolds, 2011). We used the most recent three‐year (2016–2018) mean in salmon abundance estimates prior to our plant surveys (2019) to account for interannual stochastic variation of spawning abundance.

After deriving mean spawning abundance, we calculated an average salmon density for each stream (kg/m). We replicated methods used in prior vegetation studies in the region (Hocking & Reynolds, 2011; Siemens et al., 2020; van den Top et al., 2018). Three‐year average chum and pink mean spawning abundance estimates were multiplied by the regional average weight of each species (pink = 1.05 kg, chum = 3.1 kg; Siemens et al., 2020) and divided by the length of the spawning habitat on each stream (Table S3). Salmon density ranged from 0.0 to 31.2 kg/m.

We chose salmon density (kg/m) as our salmon metric as opposed to salmon abundance (# fish) or biomass (kg). Studies in the region on mobile consumers (e.g., birds) typically use total stream biomass due to the ability of consumers to exploit resources and marine‐derived nutrients that are distributed along the whole length of the watershed (Wagner & Reynolds, 2019; Wilcox et al., 2021). Plants, however, integrate nutrients over a much smaller area. Thus, salmon density (kg/m) is a much more biologically relevant measure of nutrient availability for plants and is much more commonly used in plant‐related studies in the region (Hocking & Reynolds, 2011, Siemens et al., 2020, van den Top et al., 2018).

### 2.3 Plant field surveys

We conducted plant surveys at each stream during the summer of 2019, from July 16th to July 31st. Using a stratified random sampling approach (e.g., Hocking & Reynolds, 2011; Siemens et al., 2020), we surveyed reaches of stream proportional to 20 times the bankfull width at the river mouth, defined as the stream width at the highest tidal height (Table S3). This resulted in survey reaches between 80 and 560 m, which were subsequently divided into four strata with four randomly selected sites per stratum. At each randomly selected site, we haphazardly placed up to four 1 m^2^ quadrats as close as possible to the stream bank to sample our four study plant species.

At each quadrat, we measured several environmental covariates. First, we measured the distance of the quadrat upstream from the river mouth using a rangefinder to account for the unequal distribution of chum and pink carcasses within watersheds (Harding et al., 2019). We also measured the quadrat's distance from the stream using a transect tape as well as the average slope of each quadrat using a clinometer over a distance of 3 m, centered on each quadrat. We measured average canopy cover by using a densiometer centered on all four sides of each quadrat. Lastly, we derived measures of relative soil moisture in each quadrat. We took four measurements of soil moisture in each quadrat using a handheld soil moisture meter (FieldScout TDR 300; Spectrum Technologies, Illinois, USA), calculated an average of these readings, and subtracted this number from that day's average soil moisture at a single reference site used throughout the field season to account for variation in precipitation during our sampling period.

### 2.4 Plant measurements

We collected one leaf from each study species in each quadrat, totaling 856 plant leaves across the four species (241 false lily‐of‐the Valley, 256 salmonberry, 173 false azalea, and 186 blueberry leaves). Sample sizes were unequal due to natural variation in prevalence of each species on each stream (Hurteau et al., 2016). Leaves were then placed in a plant press for 72 h to dry, and kept in envelopes for later analysis.

We measured five plant traits as response variables: leaf nitrogen‐15, percent nitrogen, leaf mass per area, leaf area, and leaf greenness. To measure leaf nitrogen content, leaves were dried in a drying oven at 60°C for a minimum of 48 h, and ground into a fine powder using a Wig‐L‐Bug grinder. Samples were then packaged into aluminum capsules for analysis with an elemental analyzer with an isotope ratio mass‐spectrometer (EA‐IRMS). Total nitrogen content was divided by the sample weight to calculate percent nitrogen (%N), and isotopic ratios of nitrogen were calculated using the following formula: δ^15^N(‰) = (*R*_sample_/*R*_standard_–1) × 1000, where R indicates the ratio of ^15^N to ^14^N.

Prior to oven‐drying and elemental analysis, a single hole punch was taken in a consistent position from each leaf—while avoiding venation—and weighed to derive leaf mass per area (mg), or tissue density, using a Mettler‐Toledo analytical balance to a precision of 0.001 mg. Taking leaf fragments of known, consistent area allows for the quick and accurate measurement leaf mass per area when remote field site logistics may not allow for the transport of whole, fresh leaves (Pérez‐Harguindeguy et al., 2013), and has been used to measure leaf mass per area across a wide variety of taxa (Ecarnot et al., 2013; Milla‐Moreno et al., 2015; Ogaya & Peñuelas, 2007).

Increasingly, digital measurements of plant traits are being used to assess a growing array of variables using photographs from the field, as well as from satellite imagery (e.g., Gonsamo & Chen, 2014; Potithep et al., 2013; Ryu et al., 2012). This has allowed for the proliferation of field‐based measurements in remote areas, particularly when study sites are located far from laboratory facilities. This can also allow for the non‐destructive sampling of leaves and forests (Liu et al., 2017). Photographs of each leaf taken on the day of field collection were also passed through the ImageJ program to calculate “leaf greenness” and leaf area (cm^2^) (Schneider et al., 2012). Leaf greenness was calculated using the mean red, blue, and green color channel values in each image. The mean green channel value was divided by the total mean channel value of all three colors to derive a measure of leaf percent green (%). This method controls for daily variation in lighting or saturation by using the standardized amount of green in each leaf photograph (Liang et al., 2017; Tan et al., 2021; Zhang et al., 2022).

### 2.5 Statistical analyses

All statistical analyses were conducted in R (R Core Team, 2022). To assess the relationship between our five plant measurements and salmon density, we fit five separate linear and generalized linear mixed‐effects models in the lme4 and glmmTMB packages with plant species included as a fixed effect (Bates et al., 2015; Brooks et al., 2017). Each response variable was fitted as a function of salmon density, plant species and its interaction with salmon density, and our environmental covariates: quadrat distance upstream, distance from the stream, relative soil moisture, average canopy cover, and slope. We transformed (log *x* + 1) salmon density to account for its non‐linear relationship to our response variables, as this produced better model fits than untransformed salmon density. This replicated the model fits from prior work also done in the region (Hocking & Reynolds, 2011).

All continuous predictors were standardized by centering values around the mean and scaling by the standard deviation so that coefficient estimates on different scales could be compared in magnitude both within and between models (Schielzeth, 2010). A random effect of quadrat nested within stream was fit with each model to account for the non‐independence of measurements within a stream and quadrat. We used the emmeans package to calculate trends in estimated marginal means for interacting factors so that the slope of the relationship between salmon density and the response variable could be compared between plant species (Lenth, 2021).

We used the DHARMa and performance packages to verify model assumptions, check for violations in non‐collinearity of continuous predictors, test for over‐ and underdispersion, examine residual normality and homogeneity of variance, check for outliers, and verify model fit (Hartig, 2021; Lüdecke et al., 2021). Three of our models were fit with normally distributed linear mixed‐effects models (LMMs) in lme4 (nitrogen‐15, percent nitrogen, and leaf greenness models), and two of our models were fit with gamma‐distributed generalized linear mixed‐effects models (GLMMs) in glmmTMB (leaf mass per area and leaf area models). All model assumptions were validated, with the exception of evidence of heteroskedasticity in two of our models. Thus, we decided to use heteroskedasticity‐robust estimates of standard error using the clubSandwich package in models for which that calculation was possible (LMMs in lme4), and we made conservative interpretations of model coefficients with standard errors approaching zero in the remaining models (GLMMs in glmmTMB) (Pustejovsky, 2022). These models differ only marginally in the size of their standard errors and are comparable to one another.

## 3 RESULTS

### 3.1 Nitrogen‐15

**FIGURE 1:**
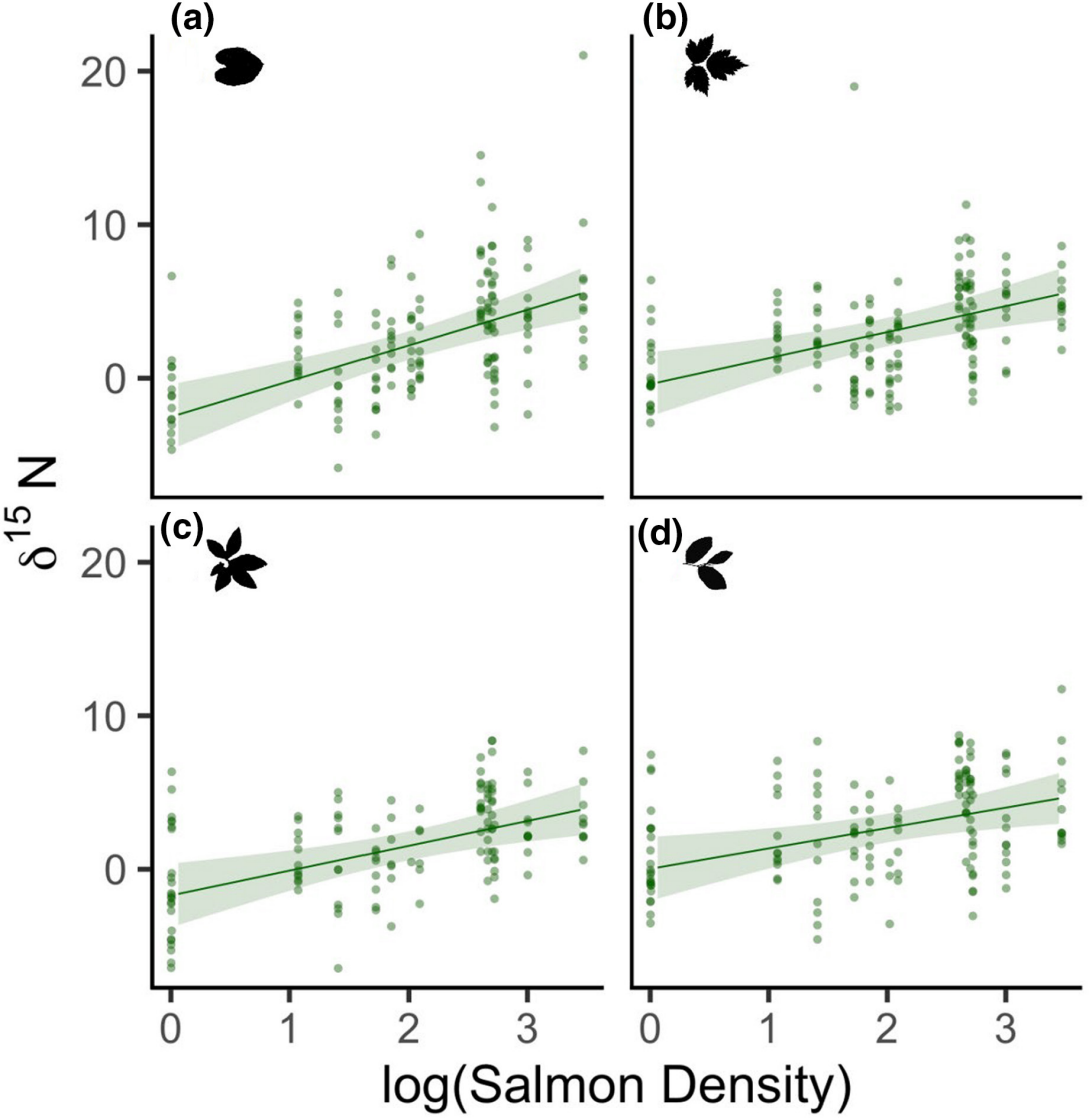
Leaf δ^15^N (‰) in (a) false lily‐of‐the‐valley, (b) salmonberry, (c) false azalea, and (d) blueberry in relation to the logarithm of salmon density (kg/m). Lines represent the generalized mixed‐effects model estimates and 95% confidence intervals using robust standard errors, while accounting for other variables in the model, and are overlaid on the raw data. *N* = 601.

As predicted, spawning salmon density had a positive relationship with the amount of nitrogen‐15 in riparian plant leaves (standardized coefficient estimate ± standard error 2.35 ± 0.49, *p* < .001; Figure 1). The effect of salmon density was more than 2.5 times stronger than any other environmental predictor. The strength of this relationship varied among plant species. False lily‐of‐the‐valley nitrogen‐15 had the strongest relationship to spawning salmon density (species‐level estimated marginal mean = 2.35 ± 0.49), followed by salmonberry (1.71 ± 0.48), false azalea (1.63 ± 0.48), and blueberry (1.34 ± 0.48). Additionally, each plant species had varying species‐specific levels of nitrogen‐15 (Table S1). Lastly, the distance upstream that a plant was growing had a negative relationship with leaf nitrogen‐15 (−0.93 ± 0.26, *p* < .001), and we found no strong evidence of an effect of relative soil moisture on leaf nitrogen‐15 (−0.30 ± 0.16; *p* = .056).

### 3.2 Percent nitrogen

**FIGURE 2:**
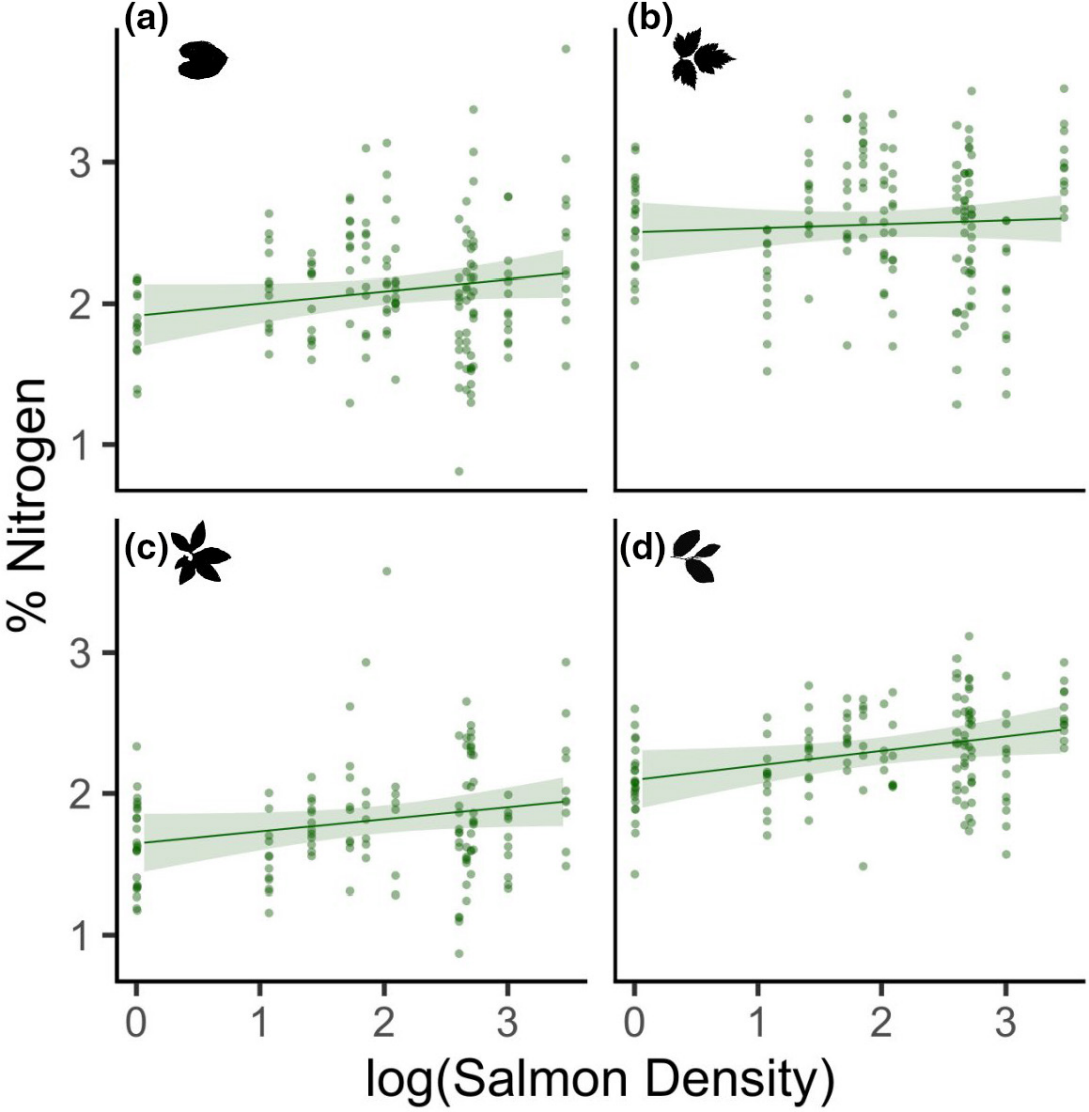
Leaf percent nitrogen (%) in (a) false lily‐of‐the‐valley, (b) salmonberry, (c) false azalea, and (d) blueberry in relation to the logarithm of salmon density (kg/m). Lines represent the linear mixed‐effects model estimates and 95% confidence intervals using robust standard errors, while accounting for other variables in the model, and are overlaid on the raw data. *N* = 601.

Contrary to our expectations, and despite having a positive relationship with nitrogen‐15, salmon density was not related to leaf percent nitrogen content (standardized coefficient estimate ± standard error 0.09 ± 0.05, *p* = .107; Figure 2). Species‐specific variation in leaf percent nitrogen (Table S1) was not related to the relationship between percent nitrogen and salmon density (all salmon density × plant species interactions N.S.; Table S1). However, plants growing in sites with higher canopy cover had higher leaf percent nitrogen content (0.06 ± 0.02, *p* = .003). For each standard deviation increase in canopy cover above the mean, there was a 0.06 SD increase in percent nitrogen.

### 3.3 Leaf mass per area

**FIGURE 3:**
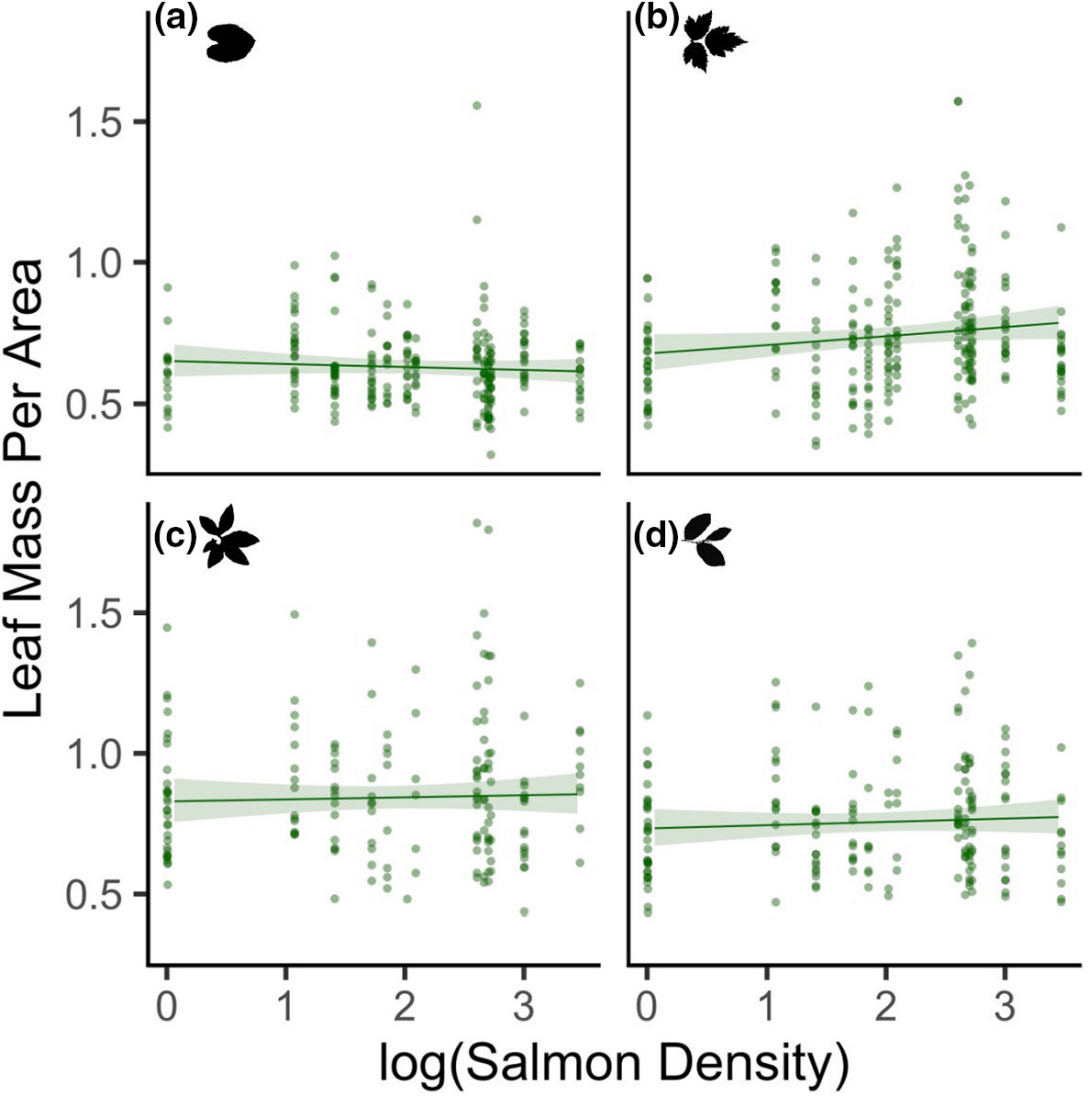
Leaf mass per area (mg) in (a) false lily‐of‐the‐valley, (b) salmonberry, (c) false azalea, and (d) blueberry in relation to the logarithm of salmon density (kg/m). Lines represent the generalized linear mixed‐effects model estimates and 95% confidence intervals, while accounting for other variables in the model, and are overlaid on the raw data. *N* = 838.

Salmon density was not related to leaf mass per area when pooled across all species (standardized coefficient estimate ± SE −0.02 ± 0.02, *p* = .41; Table S1). However, salmonberry leaves on streams with higher spawning salmon density had denser leaf tissues. (species‐level estimated marginal mean = 0.043 ± 0.02; salmon density × salmonberry coefficient = 0.06 ± 0.02, *p* = .002; Figure 3). Plants growing in sites with higher canopy cover had lower leaf mass per area (−0.08 ± 0.01, *p* < .001). For each standard deviation increase in canopy cover above the mean, there was 0.08 SD decrease in leaf mass per area.

### 3.4 Leaf greenness

**FIGURE 4:**
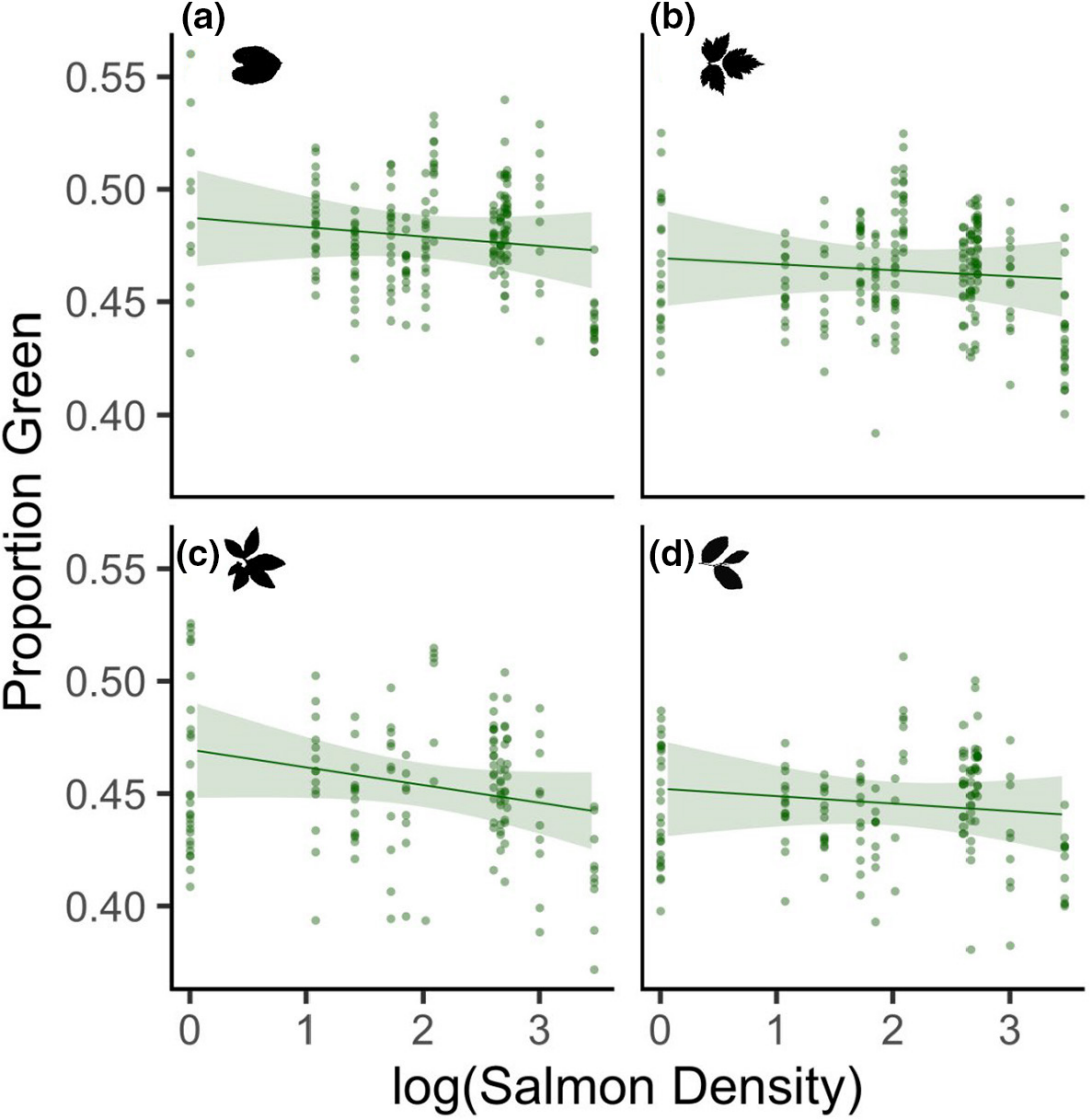
Proportion of leaf green in (a) false lily‐of‐the‐valley, (b) salmonberry, (c) false azalea, and (d) blueberry in relation to the logarithm of salmon density (kg/m). Lines represent the linear mixed‐effects model estimates and 95% confidence intervals using robust standard errors, while accounting for other variables in the model, and are overlaid on the raw data. *N* = 752.

We found no relationship between spawning salmon density and leaf greenness for any plant species (standardized coefficient estimate ± standard error – 0.00 ± 0.01, *p* = .599); all salmon density × plant species interactions N.S. (Figure 4; Table S1). There were, however, specific‐specific differences in leaf greenness among plant species (Table S1). There was a small positive relationship between plant leaf greenness and the distance the plant was found upstream (0.002 ± 0.001, *p* = .016), its distance from the stream (0.001 ± 0.000, *p* < .001), and site slope (0.002 ± 0.001, *p* < .001).

### 3.5 Leaf area

**FIGURE 5:**
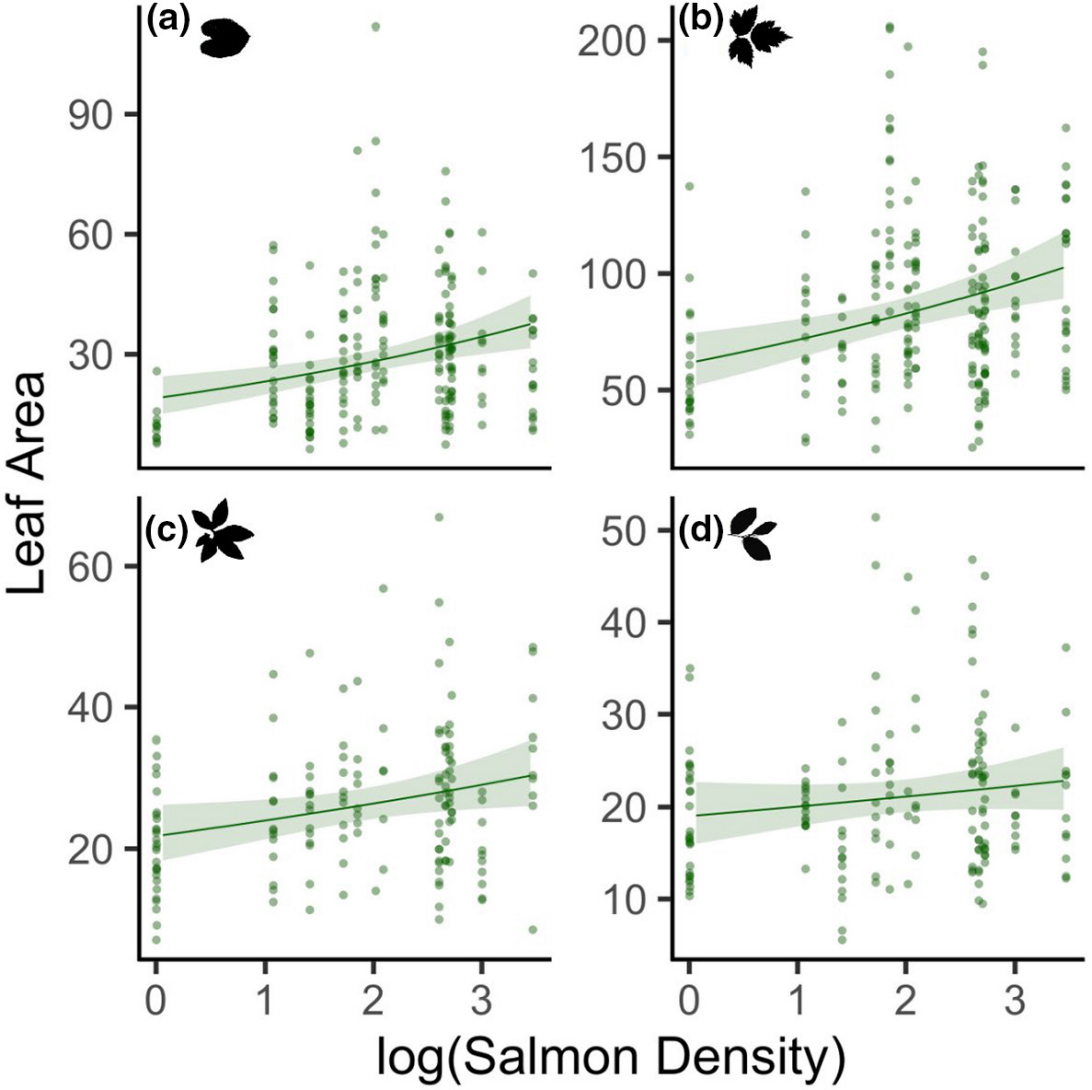
Leaf area (cm^2^) in (a) false lily‐of‐the‐valley, (b) salmonberry, (c) false azalea, and (d) blueberry in relation to the logarithm of salmon density (kg/m). Lines represent the generalized linear mixed‐effects model estimates and 95% confidence intervals, while accounting for other variables in the model, and are overlaid on the raw data. *N* = 753.

Salmon density had a positive relationship with leaf area in three of the four plant species (standardized coefficient estimate ± standard error 0.20 ± 0.06, *p* < .001; Figure 5). False lily‐of‐the‐valley leaf area had the strongest relationship to spawning salmon density (species‐level estimated marginal mean = 0.20 ± 0.06), followed by salmonberry (0.15 ± 0.04) and false azalea (0.10 ± 0.04). Blueberry leaf area did not have a significant relationship to salmon density (0.05 ± 0.04). When examining all plant species together, the effect of salmon density was nearly five times stronger than any other environmental predictor. Salmonberry had the largest leaves of the four plant species (1.01 ± 0.04, *p* < .001). Lastly, the distance from the stream that a plant was growing had a small positive relationship with leaf area (0.04 ± 0.2, *p* = .007).

## 4 DISCUSSION

Our study of 14 watersheds confirms that riparian plants appear to integrate salmon‐derived nitrogen into their foliar tissues, and may grow larger leaves on streams with more abundant salmon runs. As predicted, all four plant species growing on streams with a higher density of spawning salmon had higher levels of nitrogen‐15. However, percent nitrogen in leaf tissues did not increase with salmon density, and only salmonberry leaf tissue density increased with salmon abundance. We found no evidence that leaf greenness increases with spawning salmon density. Lastly, we found larger leaves on streams with higher spawning densities in all plant species except blueberry.

While streams with higher spawning salmon densities had higher foliar marine‐derived nitrogen in all four plant species, salmon density had no relationship with foliar percent nitrogen in these species. The nitrogen‐15 result was expected, as it has been shown in several comparisons among and within streams (Bilby et al., 2003; Hocking & Reynolds, 2011; Mathewson & Reimchen, 2003; Reimchen et al., 2002), and experimentally (Hocking & Reynolds, 2012). The relationship between salmon and foliar percent nitrogen, however, has mixed positive and null supporting evidence when examined via meta‐analysis (Walsh et al., 2020). Some prior work in our study region has shown a positive relationship between salmon density and percent nitrogen only in nitrogen indicator species such as salmonberry and false lily‐of‐the‐valley (Hocking & Reynolds, 2011), and other research showed relationships between salmon and percent nitrogen in false azalea and salmonberry using comparisons above and below waterfalls on a limited number of salmon‐bearing rivers (Mathewson & Reimchen, 2003). Conversely, experimental evidence found no relationship between salmon carcass deposition and foliar percent nitrogen in estuarine meadow plants (Dennert et al., 2023). Plant elemental composition can vary due to competition, subsidies, and nutrient limitation (Sterner & Elser, 2002), and we may only observe changes in elemental composition during excess nutrient deposition, resulting in luxury (or excess) consumption of this nutrient (Ågren, 2008; Chapin, 1980). Additionally, species with naturally high foliar nitrogen may not concentrate it in this way but increase their growth rates instead, thereby increasing the total nitrogen in their leaves rather than the percentage (Cornelissen et al., 1997). We speculate that some individual plants may not have received a large enough subsidy to release them from nutrient limitation. As chum salmon carcasses have a disproportionate effect on riparian plants due to their larger body size (Hocking & Reimchen, 2009; Siemens et al., 2020), it is possible that recent declines in regional chum returns since these prior studies may be contributing to the lack of relationship between salmon density and foliar percent nitrogen. In fact, the 5‐year mean of chum returns in the region (Pacific Fisheries Management Area 7) show a near 50% reduction when compared to the 15‐year mean, and more than a 70% reduction when compared to the 50‐year mean (Atlas et al., 2022).

We found that salmonberry leaves had a higher leaf mass per area along streams that had more salmon, yet we found no relationship between salmon density and leaf greenness. This is the opposite of what we predicted, as leaf mass per area usually increases with decreasing soil fertility and soil moisture, as plant leaves in nutrient‐poor, dry environments concentrate leaf biomass in the absence of tissue growth (Poorter et al., 2009). Leaf mass per area has also been found to vary with a variety of other processes that we were not able to control for in our study design, such as soil fertility, light intensity, temperature, season, and altitude (Poorter et al., 2009; Witkowski & Lamont, 1991). However, leaves with higher leaf mass per area tend to have a higher quantity of photosynthetic enzymes, and thus higher demand for CO_2_ (Körner & Diemer, 1987). Additionally, CO_2_ carboxylation rates in photosynthesis depend upon leaf mass per area, as well as nitrogen uptake (Iio et al., 2008). To test for possible increases in photosynthetic rate in relation to salmon density future studies should measure photosynthetic rate directly, especially in light of the previously discovered increase in salmonberry stomatal density on streams with more salmon (van den Top et al., 2018).

All plant species except blueberry had larger leaves in streams with more salmon. Larger leaf area allows for more photosynthetic infrastructure, and plant relative growth rate is closely linked to soil fertility. Moreover, leaf tissues are more likely to respond to changes in nutrient availability than other above‐ or belowground tissues (Chapin, 1980; Marschner, 1995). Prior work in the region has also found a relationship between salmon carcass deposition and flowering plant leaf size (Dennert et al., 2023), and work across the Pacific northwest has found a relationship between salmon, tree growth, and forest productivity (Brown et al., 2020; Kieran et al., 2021; Quinn et al., 2018; Reimchen & Arbellay, 2019; Reimchen & Fox, 2013).

We included ericaceous plant species in this study because we predicted that they would respond less strongly to salmon density. The lack of a leaf size effect in blueberry supports this prediction, although there was a leaf size effect in false azalea (also ericaceous). Ericaceous plants can thrive in nutrient‐poor conditions (Douglas et al., 1999b), and their association with mycorrhizal fungi often changes their relationship to soil fertility (Scagel, 2005). Ericaceous plants are more common on low salmon density streams, suggesting that they may not take advantage of high soil fertility to the same extent as their non‐ericaceous counterparts (Hocking & Reynolds, 2011; Hurteau et al., 2016). Mathewson and Reimchen (2003) also found no difference between multiple blueberry species’ percent nitrogen below and above salmon spawning barriers, yet found elevated percent nitrogen below a spawning barrier in false azalea leaves. While collectively, these findings indicate that ericaceous species are less affected by salmon‐derived nutrients than other nitrogen indicator species such as salmonberry and false lily‐of‐the valley, they also indicate that future work should include a wider range of species to better understand species‐specific variation in responses.

In conclusion, we present evidence that terrestrial plants have larger leaf area, and in some cases higher leaf mass per area, on streams with more abundant salmon populations. These increases in leaf area and tissue density, coupled with prior work demonstrating increased stomatal density (van den Top et al., 2018), indicate that riparian plants on salmon streams have access to nutrient subsidies that may increase their photosynthetic capacity. This finding could help explain why some species are found in higher abundance on salmon‐dense streams.

## AUTHOR CONTRIBUTIONS

**Allison M. Dennert:** Conceptualization (equal); data curation (equal); formal analysis (lead); investigation (equal); methodology (lead); project administration (lead); visualization (lead); writing – original draft (lead). **Elizabeth Elle:** Conceptualization (equal); funding acquisition (equal); investigation (equal); supervision (equal); writing – review and editing (equal). **John D. Reynolds:** Conceptualization (equal); funding acquisition (equal); investigation (equal); supervision (equal); writing – review and editing (equal).

## CONFLICT OF INTEREST STATEMENT

The authors declare no conflicts of interest.

## Supporting information


Appendix S1.

## Data Availability

Data and reproducible code are available on GitHub at https://github.com/adennert/salmon‐leaf‐traits. The data have been archived on Dryad at the following DOI:https://doi.org/10.5061/dryad.p5hqbzkwk
