# Supplementary material for: Nutrients from spawning salmon influence leaf area, tissue density, and nitrogen‐15 in riparian plant leaves
Source: Ecol Evol. 2024 Feb 20;14(2):e11041. doi: 10.1002/ece3.11041 (PMC10877449; doi:10.1002/ece3.11041)
Supplement: Supplementary file 1 — Appendix S1. [file ECE3-14-e11041-s001.pdf]

Appendix S1

Supplementary Material for

WORKING TITLE : Nutrients from spawning salmon influence nitrogen-15, tissue density, and area of riparian plant leaves

Table of contents

Table S1. Mixed effects model coefficients, standard errors, and model properties

Table S2. Species-level estimated marginal means for each model

Table S3. Study watershed characteristics

Figure S1. The unconstrained relationship between canopy cover, % nitrogen, and leaf mass per area

Figure S2. The constrained relationship between canopy cover, % nitrogen, and leaf mass per area

Table S1. Linear mixed effects models listed by response variable, including predictors, coefficient estimates, standard error, test statistics, and *p*-values. Generalized linear mixed-effects models list the link function associated with the error distribution. Note that the first levels of the categorical predictors are not present, as they are included in the intercept. Continuous predictors are standardized by centering around the mean and scaling by the standard deviation.

Response variable	Sample size	Distribution	Conditional R^2	Predictor variable	Estimate	SE	Test stat	*p*-value
(1) Leaf $\delta^{15}N$ (‰)	N = 601	Normal	0.714	(Intercept)	1.986	0.495	4.015	**<0.001***
				**Plant Species**				
				Salmonberry	0.880	0.341	2.582	**0.010***
				False Azalea	-0.593	0.276	-2.147	**0.032***
				Blueberry	0.571	0.238	2.404	**0.016***
				**Log salmon density**	2.349	0.489	4.805	**<0.001***
				**Distance upstream**	-0.926	0.256	-3.619	**<0.001***
				**Distance from stream**	-0.021	0.195	-0.106	0.916
				**Relative soil moisture**	-0.303	0.158	-1.912	0.056
				**Canopy Cover**	0.001	0.101	0.007	0.995
				**Slope**	-0.114	0.138	-0.823	0.410
				**Interactions**				
				Salmonberry x salmon density	-0.636	0.196	-3.239	**0.001***
				False Azalea x salmon density	-0.715	0.297	-2.403	**0.016***
				Blueberry x salmon density	-1.007	0.178	-5.671	**<0.001***
(2) Leaf percent nitrogen (%)	N = 601	Normal	0.478	(Intercept)	2.086	0.050	41.791	**<0.001***
				**Plant Species**				
				Salmonberry	0.473	0.084	5.665	**<0.001***
				False Azalea	-0.270	0.062	-4.316	**<0.001***
				Blueberry	0.216	0.065	3.315	**<0.001***
				**Log salmon density**	0.086	0.054	1.610	0.107
				**Distance upstream**	-0.006	0.026	-0.226	0.821
				**Distance from stream**	0.002	0.009	0.242	0.809
				**Relative soil moisture**	-0.006	0.017	-0.332	0.740
				**Canopy Cover**	0.058	0.020	2.978	**0.003***
				**Slope**	-0.031	0.018	-1.785	0.074
				**Interactions**				
				Salmonberry x salmon density	-0.058	0.101	-0.573	0.567
				False Azalea x salmon density	0.000	0.061	0.007	0.994
				Blueberry x salmon density	0.018	0.059	0.302	0.762

(3) Leaf mass per area (mg)	N = 838	Gamma (log link)	0.387	(Intercept)	-0.458	0.020	-23.394	**<0.001***
				**Plant Species**				
				Salmonberry	0.159	0.019	8.504	**<0.001***
				False Azalea	0.290	0.022	13.198	**<0.001***
				Blueberry	0.181	0.020	9.084	**<0.001***
				**Log salmon density**	-0.017	0.021	-0.824	0.410
				**Distance upstream**	0.009	0.011	0.823	0.410
				**Distance from stream**	-0.012	0.009	-1.301	0.193
				**Relative soil moisture**	-0.002	0.010	-0.183	0.855
				**Canopy Cover**	-0.081	0.014	-5.633	**<0.001***
				**Slope**	-0.006	0.010	-0.640	0.522
				**Interactions**				
				Salmonberry x salmon density	0.061	0.020	3.080	**0.002***
				False Azalea x salmon density	0.026	0.021	1.243	0.214
				Blueberry x salmon density	0.033	0.020	1.665	0.09598
(4) Leaf area (cm^2)	N = 753	Gamma (log link)	0.695	(Intercept)	3.335	0.047	70.550	**<0.001***
				**Plant Species**				
				Salmonberry	1.077	0.042	25.500	**<0.001***
				False Azalea	-0.064	0.046	-1.400	0.162
				Blueberry	-0.286	0.045	-6.420	**<0.001***
				**Log salmon density**	0.199	0.057	3.520	**<0.001***
				**Distance upstream**	-0.027	0.020	-1.340	0.180
				**Distance from stream**	0.040	0.015	2.720	**0.007***
				**Relative soil moisture**	-0.007	0.017	-0.430	0.669
				**Canopy Cover**	0.011	0.017	0.670	0.502
				**Slope**	0.005	0.017	0.280	0.777
				**Interactions**				
				Salmonberry x salmon density	-0.050	0.052	-0.960	0.338
				False Azalea x salmon density	0.102	0.054	-1.900	0.058
				Blueberry x salmon density	-0.145	0.053	-2.740	**0.006***

(5) Percent green (%)	N = 752	Normal	0.595	(Intercept)	0.479	0.005	93.101	**<0.001***
				**Plant Species**				
				Salmonberry	-0.015	0.002	-6.995	**<0.001***
				False Azalea	-0.025	0.002	-10.066	**<0.001***
				Blueberry	-0.033	0.003	-13.090	**<0.001***
				**Log salmon density**	-0.004	0.008	-0.525	0.599
				**Distance upstream**	0.002	0.001	2.419	**0.016**
				**Distance from stream**	0.001	0.000	4.744	**<0.001***
				**Relative soil moisture**	-0.001	0.001	-0.793	0.428
				**Canopy Cover**	0.000	0.001	-0.498	0.618
				**Slope**	0.002	0.001	3.245	**0.001***
				**Interactions**				
				Salmonberry x salmon density	0.002	0.003	0.585	0.558
				False Azalea x salmon density	-0.004	0.003	-1.205	0.228
				Blueberry x salmon density	0.001	0.003	0.275	0.783

Table S2. Species-level estimated marginal means of the linear trends in the logarithm of salmon density for each model, created using the emmeans::emtrend() function. These means are the estimated slope of the relationship between log(Salmon Density) and the response variable in each plant species. Estimated marginal means can be calculated by hand by adjusting the main salmon density coefficient by the species-level interaction coefficients. Note that these standard errors are not presented as heteroscedasticity-robust standard errors; thus, confidence intervals that come close to zero must be interpreted conservatively.

Response Variable	Plant Species	Est. Marginal Mean	SE	Lower 95% CI	Upper 95% CI
(1) Leaf $\delta^{15}N$ (‰)	False lily-of-the-valley	2.35	0.489	1.310	3.39
	Salmonberry	1.71	0.483	0.682	2.74
	False azalea	1.63	0.482	0.603	2.66
	Blueberry	1.34	0.480	0.314	2.37
(2) Leaf percent nitrogen (%)	False lily-of-the-valley	0.086	0.051	-0.019	0.191
	Salmonberry	0.028	0.049	-0.074	0.130
	False azalea	0.087	0.049	-0.015	0.188
	Blueberry	0.104	0.049	0.004	0.205
(3) Leaf mass per area (mg)	False lily-of-the-valley	-0.017	0.021	-0.058	0.024
	Salmonberry	0.043	0.022	0.000	0.087
	False azalea	0.009	0.023	-0.036	0.053
	Blueberry	0.016	0.022	-0.027	0.058
(4) Percent green (%)	False lily-of-the-valley	-0.004	0.005	-0.015	0.007
	Salmonberry	-0.003	0.005	-0.013	0.008
	False azalea	-0.008	0.005	-0.019	0.003
	Blueberry	-0.003	0.005	-0.014	0.007
(5) Leaf area (cm^2)	False lily-of-the-valley	0.199	0.057	0.088	0.310
	Salmonberry	0.149	0.042	0.066	0.232
	False azalea	0.097	0.043	0.012	0.182
	Blueberry	0.054	0.042	-0.029	0.136

Table S3. Stream names, locations, habitat characteristics, and salmon density data for each watershed included in this study.

**Stream Name**	**Location (decimal degrees)**	**Watershed Area (km^2)**	**Spawning Length (m)**	**Salmon Density (kg/m)**	**Bankfull Width (m)**
Lee	52.5129, -127.8321	11.2	800	6.54	16.9
Farm Bay	52.2333, -128.0329	2.3	0	0.00	15.2
Quartcha	52.5149, -127.8405	29.4	5500	14.21	20.0
Bullock Main	52.4029, -128.0785	3.3	540	13.37	8.8
Clatse	52.3377, -127.8391	24.3	835	31.17	28.0
Hooknose	52.1249, -127.8370	14.8	1800	1.93	15.3
Goatbushu	52.2132, -127.8767	4.5	550	4.59	6.8
Sagar	52.0959, -127.8388	36.6	180	19.15	11.0
Fancy Right	52.0585, -128.0167	9.9	325	3.10	6.2
Jane	52.0505, -128.0607	1.3	0	0.01	3.3
Kunsoot	52.1490, -128.0078	4.9	900	12.54	15.1
Fannie	52.0426, -128.0668	16.4	1375	13.90	23.3
Kill	52.4260, -128.0950	0.5	420	5.37	6.3
Beales Left	52.1877, -127.9832	6.5	235	7.09	12.1

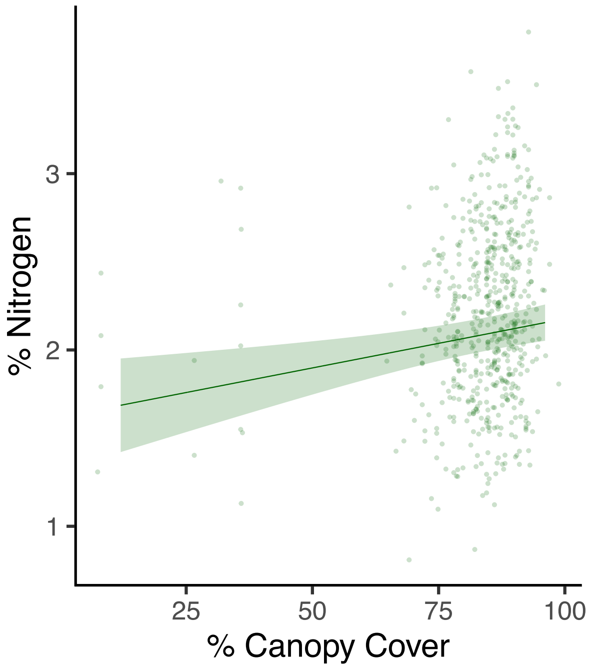

Figure S1. Leaf percent nitrogen (%) and leaf mass per area (mg) in response to percent canopy cover (%). Lines represent the linear and generalized linear mixed-effects model estimates and 95% confidence intervals, while accounting for other variables in the model, and are overlaid on the raw data. N = 601 (left), N = 838 (right).

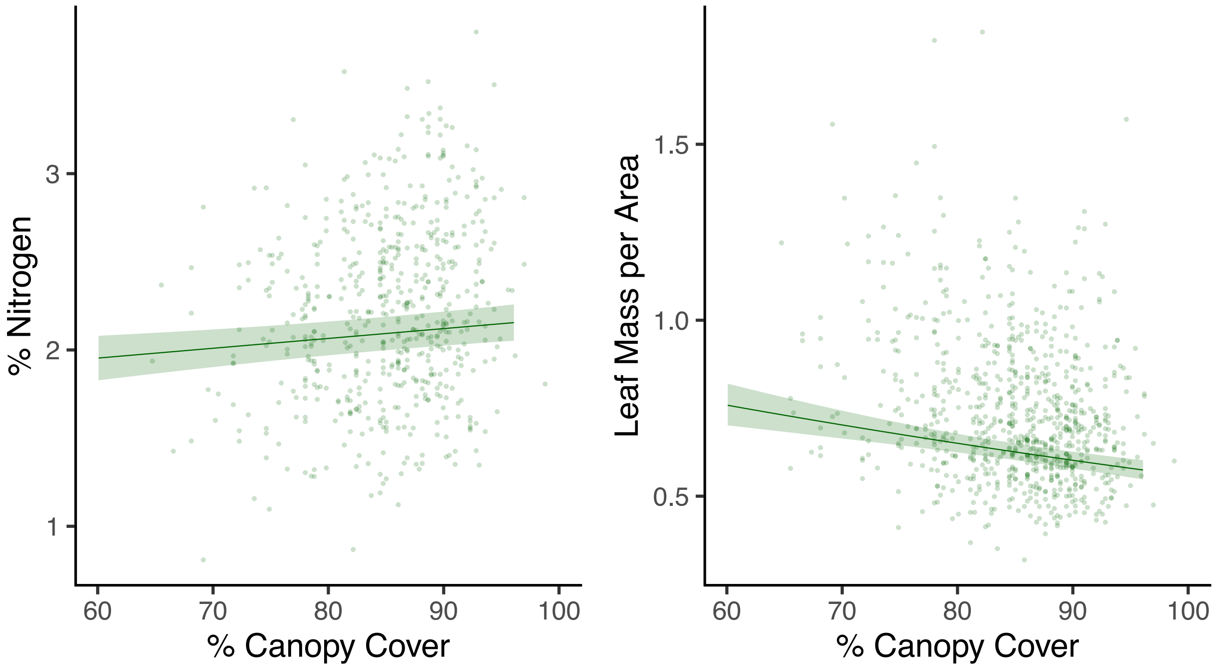

Figure S2. Leaf percent nitrogen (%) and leaf mass per area (mg) in response to percent canopy cover (%). Lines represent the linear and generalized linear mixed-effects model estimates and 95% confidence intervals, while accounting for other variables in the model, and are overlaid on the raw data. N = 601 (left), N = 838 (right). Due to strong left skew in the distribution of the canopy cover data, canopy cove values lower than 60% have been removed from the figure for ease of display (approx. 20 values in each panel).
